# Advancing Precision Rehabilitation Through a Sensor-Based 6-DoF Robotic Exoskeleton: Clinical Validation and Ergonomic Assessment

**DOI:** 10.3390/s26010088

**Published:** 2025-12-23

**Authors:** Hande Argunsah, Begum Yalcin, Mehmet Alper Ergin, Gokay Coruhlu, Mustafa Yalcin, Volkan Patoglu, Zeynep Guven

**Affiliations:** 1 Department of Biomedical Engineering, Faculty of Engineering and Natural Sciences, Acibadem Mehmet Ali Aydinlar University, Istanbul 34752, Turkey; 2 Department of Biomedical Engineering, Graduate School of Natural and Applied Sciences, Acibadem Mehmet Ali Aydinlar University, Istanbul 34752, Turkey; begum.yalcin@acibadem.edu.tr; 3 Faculty of Engineering, Natural Sciences of Sabancı University, Istanbul 34956, Turkey; aergin@alumni.sabanciuniv.edu (M.A.E.); gokaycoruhlu@alumni.sabanciuniv.edu (G.C.); myalcin@alumni.sabanciuniv.edu (M.Y.); volkan.patoglu@sabanciuniv.edu (V.P.); 4 Department of Physical Medicine and Rehabilitation, School of Medicine, Acibadem Mehmet Ali Aydinlar University, Istanbul 34752, Turkey; zeynep.guven@acibadem.edu.tr

**Keywords:** sensor-based rehabilitation, upper-extremity exoskeleton, human–robot interaction, ergonomic design, clinical validation, kinematic assessment, assistive robotics

## Abstract

**Highlights:**

**What are the main findings?**

- The self-aligning 6-DoF robotic exoskeleton accurately replicated upper-extremity kinematics across passive and active modes, confirming reliable motion tracking and ergonomic alignment.
- Clinical pilot testing in patients with shoulder impairments demonstrated high usability, comfort, and repeatability across all movement tasks.

**What are the implications of the main findings?**

- These results validate the feasibility of sensor-based robotic systems for objective assessment and personalized upper-limb rehabilitation.
- The study supports the integration of self-aligning exoskeletons into precision rehabilitation frameworks to enhance patient recovery and clinical decision-making.

**Abstract:**

Effective upper-extremity rehabilitation requires intensive and precise movement training, yet conventional therapies lack accurate motion tracking. Robotic exoskeletons address this limitation but are often hindered by ergonomic misalignment and limited adaptability. The AssistOn-Arm, a novel self-aligning exoskeleton, integrates ergonomic design and back-drivable actuation to enhance comfort and facilitate natural user interaction. This study aimed to assess the usability and ergonomics of the device in healthy participants and to conduct a pilot clinical evaluation in individuals with upper-extremity impairments. Thirty healthy participants and twelve patients with shoulder impairments performed predefined tasks under participant-active and device-active conditions. Kinematic data captured concurrently with AssistOn-Arm and Xsens MVN demonstrated strong agreement between conditions. Quantitative analysis revealed no significant differences (*p* > 0.05) in flexion, elevation, abduction–adduction, and external rotation, indicating reliable alignment with natural joint axes. Significant differences (*p* < 0.05) were observed only in sagittal hyperextension and internal rotation, reflecting device mechanical constraints. The study confirms the clinical feasibility of AssistOn-Arm as a sensor-driven, self-aligning exoskeleton that bridges engineering innovation and precision rehabilitation, paving the way for its integration into clinical practice.

## 1. Introduction

The shoulder girdle is the most mobile joint complex in the human body, enabling the upper extremity to position the hand for functional tasks. Its intricate biomechanical interactions facilitate movements across multiple planes, making it one of the most challenging regions to rehabilitate. Impairments in shoulder function—such as reduced range of motion (ROM), muscle weakness, and instability—can arise from musculoskeletal, neurological, or rheumatological conditions [1,2,3,4,5]. These impairments frequently limit the performance of activities of daily living (ADLs) [6], creating the need for effective short- and long-term rehabilitation strategies. The primary goal of upper-extremity rehabilitation is to restore strength, ROM, stability, and functional capacity, thereby improving patients’ quality of life. Evidence shows that higher daily intensity and repetition of rehabilitation activities lead to superior outcomes [7]. However, current rehabilitation protocols—particularly in neurological populations—lack consensus on optimal regimens, and clinical assessments often rely on subjective measures. Robotic rehabilitation devices address these limitations by offering objective, quantitative assessments of motor performance, enabling precise documentation of functional changes and allowing clinicians to adapt treatment plans in a reproducible and patient-specific manner.

Exoskeletons with multiple degrees of freedom (DoF) at the shoulder and elbow are especially important for supporting ADLs such as eating, drinking, and reaching. These devices provide a greater ROM and adaptability by distributing torque appropriately across joints and muscles [8,9,10,11]. Yet, given the anatomical complexity of the shoulder, robotic exoskeletons must ensure accurate joint alignment to prevent internal stresses. Devices with self-aligning kinematics have therefore been proposed to accommodate natural shoulder motion, improving ergonomics and user comfort across the full ROM.

This study introduces the AssistOn-Arm, a novel 6-DoF self-aligning robotic rehabilitation device, which combines advanced kinematic architecture, impedance-based control, and quantitative diagnostic capabilities to support both therapeutic training and motion assessment. The AssistOn-Arm exoskeleton was developed within the context of academic research on self-aligning upper-limb rehabilitation devices [12,13,14]. The system builds upon earlier shoulder–elbow exoskeleton concepts and has been refined as a modular, sensor-integrated platform to support both kinematic assessment and active rehabilitation. The present study evaluates this research prototype in terms of ergonomic alignment, usability, and clinical feasibility. Its product development emphasized safety, ergonomic usability, and adaptability to anatomical variability, while its evaluation involved systematic testing in both healthy participants and patients with shoulder impairments. Beyond validating the device in healthy individuals, a pilot clinical study was conducted in a patient cohort to explore its feasibility and applicability. This pilot investigation provided preliminary insights into how the device accommodates pathological movement patterns, preserves natural kinematics, and delivers reliable quantitative metrics during therapeutic tasks. We hypothesize that the AssistOn-Arm supports safe and effective rehabilitation, maintains natural movement strategies across both healthy and impaired populations, and offers robust quantitative assessments, thereby addressing key limitations of traditional rehabilitation approaches and advancing its potential for clinical adoption.

## 2. Materials and Methods

### 2.1. Participants

Thirty healthy volunteers (13 females, 17 males; mean age 25.4 ± 4.8 years; mean height 1.61 ± 0.05 m; mean mass 68.8 ± 15.9 kg; mean BMI 23.9 ± 2.99 kg/m^2^) were recruited. Eligibility criteria for inclusion were as follows: (1) Age between 18 and 60 years; (2) Right-hand dominance; (3) No history of neuromuscular, rheumatological, or orthopedic disorders affecting motor control; (4) No prior upper-extremity surgery or trauma resulting in lasting impairment; (5) For the patient group, a clinical diagnosis of shoulder pathology confirmed by an orthopedic specialist (e.g., impingement, rotator cuff tear, labral injury, or recurrent dislocation); (6) Ability to understand and follow verbal instructions; (7) Absence of contraindications for robotic rehabilitation (e.g., open wounds, severe pain, or unstable fractures). All were right-hand dominant.

The patient group included 12 individuals with clinically diagnosed shoulder impairments (5 females, 7 males; mean age 45.8 ± 11.2 years; mean height 1.65 ± 0.12 m; mean mass 73.1 ± 10.2 kg; mean BMI 27.4 ± 1.36 kg/m^2^) (Table 1). Eligibility criteria for inclusion were as follows: (1) Age between 18 and 70 years; (2) Unilateral or bilateral shoulder impairment of orthopedic origin (e.g., subacromial impingement, rotator cuff tear, labral lesion, or recurrent instability) clinically confirmed by a licensed physician; (3) Presence of pain, reduced range of motion, or functional limitation requiring rehabilitation intervention; (4) Stable medical condition and ability to participate in robotic training without cardiopulmonary contraindications; (5) Intact cognitive function and ability to understand and follow verbal instructions; (6) No history of neurological disorders (e.g., stroke, multiple sclerosis, Parkinson’s disease) or systemic rheumatological disease affecting upper-limb control; (7) No open wounds, skin irritation, or recent surgical incisions at the shoulder or upper limb; (8) Absence of implanted medical devices or metal implants that could interfere with exoskeleton operation or motion tracking. Exclusion criteria included: (1) Severe shoulder pain preventing active or passive movement; (2) Acute infection or inflammation of the shoulder region; (3) Inability to tolerate the exoskeleton interface or follow the testing protocol. Diagnoses included subacromial impingement, rotator cuff tear, labral injury, and recurrent dislocation. Although individual diagnoses varied, all patients presented with orthopedic shoulder impairments characterized by pain, reduced ROM, and functional limitations requiring rehabilitation. Therefore, the group was considered homogeneous in terms of clinical presentation and therapeutic needs. Healthy participants were recruited through public announcements and university mailing lists between May and September 2019. The patient group was recruited from the Department of Physical Medicine and Rehabilitation between October 2019 and February 2020. Eligible individuals were screened based on the inclusion criteria, and all participants provided written informed consent prior to participation. The study was conducted in accordance with the Declaration of Helsinki, and the protocol was approved by the Ethics Committee of Acibadem University (Project identification code: 2018-7/2).

### 2.2. Self-Aligning Upper-Extremity Rehabilitation Device: AssistOn-Arm

AssistOn-Arm (Interact Technologies, Istanbul, Turkey) is a powered robotic exoskeleton developed to support intensive, task-specific rehabilitation of the upper extremity [12]. Its design addresses the need for high-repetition, goal-directed exercises that promote neuroplasticity and functional recovery in patients with neurological, orthopedic, or musculoskeletal impairments.

**Figure 1:**
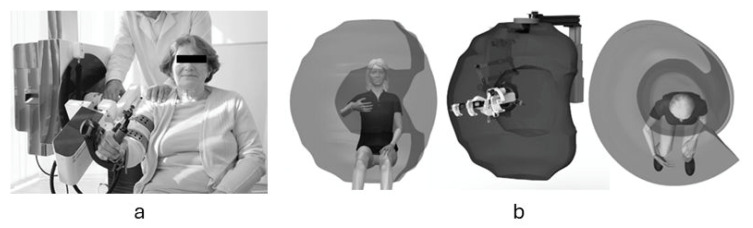
(**a**) Clinical application of the AssistOn-Arm under therapist supervision. The device’s self-aligning exoskeletal structure enables safe guidance of upper-limb movements. (**b**) Three-dimensional workspace envelope of the AssistOn-Arm showing reachable positions in frontal, sagittal, and transverse planes. The hemispherical volume corresponds to the natural motion of the human shoulder, confirming broad kinematic coverage and absence of operational singularities within the therapeutic range.

The device provides six DoF, five active DoFs at the shoulder complex and one at the elbow joint. This configuration enables replication of natural arm kinematics and supports the wide range of functional movements required for ADLs. The shoulder module integrates multiple axes of rotation to reproduce complex glenohumeral and scapulothoracic motions. A key innovation is its self-aligning joint architecture, which continuously adapts to user movement, maintaining proper alignment between human and device joint axes. This mechanism is designed to reduce internal stresses, limit the risk of misalignment, and support ergonomic movement throughout the physiological range of motion, which is particularly important for effective shoulder rehabilitation (Figure 1).

The operational workspace of the AssistOn-Arm was quantified through CAD-based kinematic simulation and verified experimentally. The exoskeleton enables a total reachable volume of approximately 0.45 m^3^, encompassing 95% of the physiological range of shoulder and elbow motions. Table 2 summarizes the angular and translational range of each anatomical plane compared with typical human joint mobility. The system supports coordinated motion across frontal, sagittal, horizontal, and scapular planes, reproducing the natural hemispherical workspace of the upper limb [13].

The device architecture minimizes kinematic singularities through its self-aligning shoulder module, which employs parallel-axis linkages to maintain instantaneous center of rotation alignment with the glenohumeral joint. Singularities that could occur at extreme ranges of motion are mechanically avoided through workspace constraints that ensure stable torque transmission across the full operating range. These design constraints prevent internal stresses while preserving functional workspace for daily activities and rehabilitation tasks. Details of kinematic and singularity analyses of AssistOn-Arm exoskeleton and their experimental verification are available in [15].

To enable natural human–robot interaction, AssistOn-Arm employs an impedance-based control strategy that modulates joint torques in response to patient effort and external forces [12,13]. This allows voluntary movement with minimal interference while providing adjustable assistance or resistance as required. The gravity-compensation module decreases the effective load on the limb, facilitating movement in individuals with reduced muscle strength or tone and enhancing the user’s ability to contribute actively to the movement [7,8,16,17]. Beyond movement support, the device was designed to minimize compensatory strategies by isolating joint motion and promoting correct movement patterns. This therapeutic focus is complemented by embedded sensors that record high-resolution kinematic and kinetic data, including joint angles, forces, torques, ROM, velocity, and acceleration. These features transform AssistOn-Arm into both a training and diagnostic tool, offering objective, reproducible metrics for patient monitoring. By integrating assessment capabilities directly into the device, the need for external motion capture systems or force platforms is reduced, improving clinical feasibility and streamlining data collection.

### 2.3. Experimental Design and Procedure

A standardized testing protocol was developed and applied to all participants, covering fundamental shoulder joint movements. Kinematic data were recorded using the Xsens MVN motion capture system (Movella, Enschede, The Netherlands) (Table 3). The study was conducted in two sequential stages:

Stage 1—Ergonomics and Usability Tests (Healthy Participants): (a) Familiarization with the AssistOn-Arm and safety briefing (b) Baseline kinematic recording using Xsens MVN during free movement (c) Participant-Active condition: the device operated in passive mode while participants performed voluntary movements across all nine standardized tasks (M1–M9) (d) Device-Active condition: the exoskeleton replayed recorded trajectories while participants remained passive (e) Simultaneous kinematic recording with both Xsens MVN and AssistOn-Arm sensors for cross-validation (f) Post-session questionnaire on comfort, alignment, and perceived effort. After completing each session, participants completed a 13-item usability and discomfort questionnaire designed to assess ease of connection, freedom of movement, safety, and post-session comfort. Each item was rated on a 5-point Likert scale (1 = strongly disagree, 5 = strongly agree). The complete questionnaire and corresponding item statistics are provided in Supplementary Material S2.

Stage 2—Patient Tests (Shoulder-Impaired Participants): (a) Clinical examination and confirmation of eligibility by a rehabilitation physician (b) Familiarization with the device and adjustment of the exoskeleton to the patient’s anthropometry (c) Participant-Active condition only: patients performed voluntary movements for tasks M1–M6 and M9 (movements M7–M8 were omitted due to risk of over-extension) (d) Real-time data capture using the AssistOn-Arm sensors for joint angles, torques, and ROM (e) Monitoring for safety and comfort throughout each trial (f) Immediate post-session documentation of subjective feedback and motion repeatability.

Movements 7 and 8 require extreme ranges and involve multi-planar coordination, which could introduce compensatory patterns in patients with shoulder impairments. Their omission allowed the study to focus on reproducible, anatomy-based tasks, providing a clearer foundation for evaluating the device’s feasibility and accuracy. Flexion, elevation, and abduction–adduction are essential for overhead and lateral reaching, dressing, and lifting activities. Hyperextension and behind-trunk rotations reflect functional tasks such as reaching the back or fastening clothing, which are often restricted in patients with shoulder impairments. Internal and external rotations are critical for joint stability and common ADLs such as grooming, eating, and tool use. Horizontal adduction–abduction simulates cross-body reaching, necessary for self-care and feeding. Together, these tasks provide a comprehensive assessment of the functional ROM, compensatory tendencies, and the capacity of AssistOn-Arm to reproduce complex multi-planar shoulder mechanics in a clinically meaningful manner.

Prior to testing, all participants were introduced to the AssistOn-Arm and briefed on the study’s purpose, objectives, and procedures. Safety instructions were provided, including a demonstration of the device’s key features and the use of the emergency stop button, which participants were instructed to activate in case of discomfort or perceived risk. Before each testing session, participants were provided with a standardized demonstration of the AssistOn-Arm’s operating modes, safety functions, and emergency procedures under direct therapist supervision. A summary of this standardized demonstration and emergency management protocol is provided in Supplementary Material S1. During each trial, kinematic data were recorded concurrently with the Xsens MVN and the AssistOn-Arm. The Xsens MVN served as an external reference to ensure high-accuracy measurements in both free-movement and exoskeleton-assisted conditions [17,18,19]. By capturing a standardized set of predefined movement tasks, the protocol allowed for a detailed assessment of upper-extremity kinematics, repeatability, and ergonomic performance of the AssistOn-Arm.

### 2.4. Statistical Analysis

Continuous variables were summarized as mean ± standard deviation (SD). To evaluate differences in ROM across experimental conditions, repeated-measures analyses of variance (ANOVA) were conducted separately for each movement task (M1–M9). Within-subject factors included condition (Participant-Active [PA], Device-Active [DA], and Free Movement), allowing assessment of whether AssistOn-Arm measurements aligned with Xsens MVN data and natural movement patterns. For each ANOVA, the sum of squares, mean square, F-statistic, degrees of freedom (df), and significance level (σ) were computed and reported in Table 4. When significant main effects were identified (*p* < 0.05), post hoc comparisons with Bonferroni correction were applied to localize differences between specific conditions. This approach provided a robust evaluation of the extent to which the exoskeleton replicated natural shoulder kinematics under both active participation and device-driven execution. For all inferential analyses, *p* < 0.05 was considered statistically significant. Effect sizes were reported as partial η^2^ for ANOVA outcomes and Cohen’s d with 95% confidence intervals for pairwise comparisons, providing information on the magnitude and practical relevance of observed differences. The usability and discomfort questionnaire results were summarized as mean ± standard deviation for descriptive purposes. All statistical analyses were performed using IBM SPSS Statistics (version 21; IBM Corp., Armonk, NY, USA).

## 3. Results

The evaluation of AssistOn-Arm was structured into two main stages: Ergonomics and Usability Tests and Patient Tests. The first stage focused on healthy participants, allowing systematic assessment of ergonomics, usability, and measurement accuracy under both participant-active and device-active conditions, with simultaneous data collection using Xsens MVN and the AssistOn-Arm. This phase enabled validation of the device’s ability to reproduce natural movement patterns, maintain joint alignment, and ensure user comfort across a range of functional tasks. The second stage involved patient tests, which were designed to explore the feasibility and safety of AssistOn-Arm in a clinical context. In this stage, individuals with orthopedic shoulder impairments performed participant-active, and kinematic data were collected exclusively through the embedded sensors of AssistOn-Arm. This approach provided an initial examination of the device’s clinical applicability and its capacity to capture relevant motor performance parameters in patient populations.

### 3.1. Ergonomics and Usability Test Results

Table 4 presents ROM outcomes and repeated-measures ANOVA statistics across nine movement tasks. In shoulder flexion (M1) and elevation (M2), AssistOn-Arm values closely matched Free Movement, with mean differences < 10°. ANOVA indicated significant differences (M1: F = 9.01, *p* < 0.05; M2: F = 4.52, *p* = 0.014), but effect sizes were moderate (d = 0.62 [0.31, 0.98] for flexion; d = 0.78 [0.50, 1.17] for elevation), supporting strong reproducibility of natural trajectories. Shoulder hyperextension (M3) also showed consistent results, with non-significant ANOVA (*p* = 0.090) and small-to-medium effect sizes (d = 0.39 [0.03, 0.76] vs. Xsens MVN; d = −0.60 [−1.06, −0.24] vs. Free), indicating minor but systematic offsets attributable to workspace limits.

More pronounced deviations emerged in multi-planar and rotational movements. Abduction–adduction (M4) showed significant differences (F = 9.36, *p* < 0.05), with large effects against Xsens (d = −1.06 [−1.59, −0.70]) but smaller differences against Free (d = −0.72 [−1.21, −0.37]). External rotation at 90° abduction (M5) exhibited very large effects (d = −1.47 [−2.20, −1.17]) despite consistent alignment with Free Xsens MVN measurements. Internal/external rotation beside the trunk at 90° elbow flexion (M6) revealed strong effects (d = −0.90 [−1.59, −0.48] vs. Xsens MVN; d = −0.54 [−1.23, −0.05] vs. Free Measurement), underscoring the challenges of replicating trunk-adjacent rotations. The largest discrepancies occurred in overhead and behind-the-back movements (M7, M8), where very large effect sizes (d up to −2.52) reflected substantial constraints in ROM compared with Free Movement. Horizontal adduction–abduction (M9) produced ROM values closely aligned across conditions, with no significant ANOVA result (*p* = 0.081). Nevertheless, large effects were observed between AssistOn-Arm and Free Xsens MVN measurements (d = 0.93 [0.60, 1.39]), indicating systematic but modest offsets that did not reach statistical significance.

Following each testing session, participants completed a 13-item usability and discomfort questionnaire (Supplementary Material S2). The questionnaire demonstrated excellent internal consistency (Cronbach’s α = 0.97). Overall, responses indicated a high level of perceived safety and ease of use. Participants agreed that device connections were easily installed and removed (mean = 4.35 ± 0.81 and 4.45 ± 0.60, respectively) and that the handle was easy to use (4.29 ± 0.47). Minimal discomfort or irritation was reported during or after testing (Q4 = 4.60 ± 0.68; Q12 = 4.70 ± 0.47; Q13 = 4.45 ± 0.76). Ratings related to movement freedom and stress (Q8 = 3.70 ± 0.66; Q9 = 4.05 ± 0.89) suggest that the device supported functional motion without substantial restriction. Collectively, these findings confirm high user acceptability and ergonomically safe interaction with the AssistOn-Arm during both healthy and patient trials.

### 3.2. Pilot Test Results

**Figure 2:**
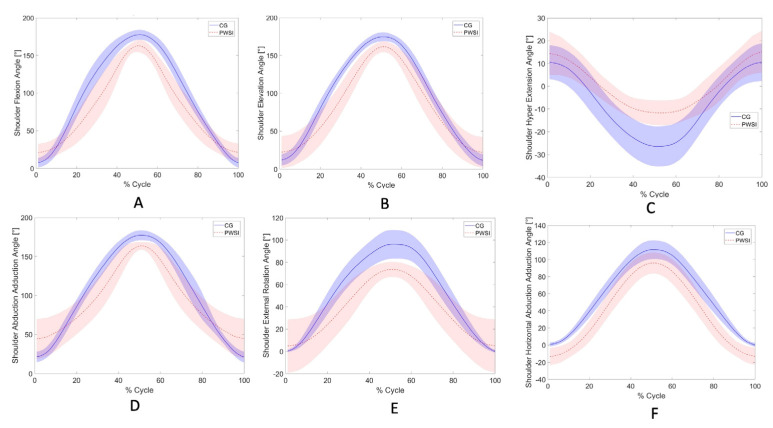
Shoulder kinematic profiles of anatomically standardized ADL recorded with the AssistOn-Arm during participant-active conditions. Data are shown for healthy controls (CG, solid blue line, shaded 95% CI) and patients with shoulder impairment (PWSI, dashed red line, shaded 95% CI). Each subplot represents the mean joint angle trajectory across the normalized movement cycle (0–100%): (**A**) Shoulder flexion, (**B**) Shoulder elevation, (**C**) Shoulder hyper-extension, (**D**) Shoulder abduction–adduction, (**E**) Shoulder external rotation, and (**F**) Shoulder horizontal abduction–adduction.

Twelve patient participants successfully completed the study under Participant-Active mode. Upper-extremity kinematics during standardized movement tasks (M1–M6, M9) performed across multiple anatomical planes were recorded exclusively with the AssistOn-Arm, enabling evaluation of its ergonomic feasibility, workspace coverage, and functional applicability in clinical use (Figure 2).

## 4. Discussion

The results indicated that the AssistOn-Arm reproduced upper-limb kinematics with a high degree of repeatability and ergonomic alignment, comparable to reports from other exoskeleton systems designed for shoulder rehabilitation [7,8,17]. The consistent replication of multi-planar motions across both participant-active and device-active conditions supports the mechanical adaptability of its self-aligning joint structure. These kinematic findings were further supported by the usability questionnaire, in which participants reported minimal discomfort and high perceived safety during all test conditions. Nevertheless, deviations observed during complex rotational tasks (M5–M8) suggest that mechanical constraints at extreme ranges may limit the full expression of physiological motion. Such limitations have also been noted in comparable shoulder exoskeletons, where joint coupling and linkage geometry restrict end-range rotations to maintain user safety and mechanical stability [10,11].

From a clinical standpoint, the device effectively maintained scapulohumeral rhythm and allowed participants to reach functional ranges of motion without discomfort, supporting its ergonomic suitability for early to mid-stage rehabilitation. However, future versions should aim to enhance rotational workspace and fine-tune impedance parameters to better accommodate individualized patient needs. The close correspondence between participant-active and device-active outcomes further indicates that the record-and-play functionality can reliably reproduce natural movement trajectories, which may facilitate task-specific motor training and clinician-supervised progression. Overall, while these findings validate the system’s usability and kinematic reliability, ongoing optimization and longitudinal trials are warranted to determine its long-term therapeutic impact and comparative efficacy against existing robotic rehabilitation platforms.

Pilot study outcomes indicated that patients were able to complete all functional shoulder movements within the designed workspace of the device. Only sagittal plane hyperextension showed reduced values, which were attributable to intentional mechanical constraints of the AssistOn-Arm to ensure user safety. For all other movements, no significant restrictions were observed, indicating that the device did not hinder natural kinematics when operating within its functional range. These findings collectively suggest that AssistOn-Arm can be safely and effectively used in patients with shoulder impairments, offering reproducible movement guidance and reliable capture of kinematic performance without compromising comfort or clinical usability.

This study has following limitations that should be acknowledged. First, the number of participants, particularly within the patient group, was relatively small. A larger cohort including individuals with a broader spectrum of shoulder impairments would strengthen the robustness of the results and enhance the generalizability of the findings. Second, the study focused only on short-term usability; although participants reported no discomfort during testing, long-term wearability, potential fatigue, and sustained ergonomic comfort were not assessed. Future studies should evaluate device performance and user experience over extended sessions to better capture its long-term clinical applicability. Third, while the device enabled most shoulder movements, mechanical constraints limited extreme ranges, particularly hyperextension in the sagittal plane. Future design iterations should aim to expand the ROM to better accommodate functionally demanding tasks.

## 5. Conclusions

These findings collectively suggest that the AssistOn-Arm can be safely applied in patients with shoulder impairments, providing consistent movement guidance and accurate kinematic measurements while maintaining user comfort and clinical practicality. The device enabled safe execution of major shoulder movements with negligible reported discomfort, as supported by post-session questionnaire results, and provided reliable quantitative motion data. The device demonstrated only minor restrictions in extreme hyperextension and provided reliable quantitative motion data. These results suggest that AssistOn-Arm can deliver effective task-specific rehabilitation through repetitive passive and active training while maintaining ergonomic alignment and measurement accuracy. Further studies with larger and more diverse patient populations are warranted to confirm its clinical effectiveness and long-term usability.

## 6. Patents

Volkan Patoglu holds patent US20120330198A1, issued to Volkan Patoglu.

## Figures and Tables

**Table 1:** Diagnosis of the Patients with Shoulder Impairments.

Diagnostic Category	Specific Diagnosis (n)	Sex (M/F)	Total (n)
Impingement Syndromes	Subacromial (6); Acromioclavicular (1)	5/3	7
Rotator Cuff Pathology	Rotator cuff tear (2)	1/1	2
Labral Pathology	SLAP tear (1)	1/0	1
Instability Disorders	Recurrent dislocation (1)	0/1	1
Degenerative Changes	Osteoarthritis, Grade 1 (1) *	0/1	1

* n = number of patients in each specific diagnosis.

**Table 2:** Human shoulder ROM compared to the AssistOn-Arm exoskeleton active operational workspace.

Joint Plane	Movement	ROM (Amplitude/Range)
Frontal	Abduction/Adduction	Human: 180°/30° (Range 210°) AssistOn-Arm: 179°/30° (Range 209°)
Sagittal	Flexion/Extension	Human: 180°/50° (Range 230°) AssistOn-Arm: 179°/50° (Range 229°)
Horizontal	Abduction/Adduction	Human: 30°/140° (Range 170°) AssistOn-Arm: 30°/140° (Range 170°)
Scapular	Elevation/Depression, Protraction/Retraction	Human: 30/50 mm, 25/25 mm (Range 80 mm, 50 mm) AssistOn-Arm: 120/120 mm (Range 240 mm)

Amplitude values represent maximum angles or linear displacements achievable in each movement direction. Range denotes the total achievable motion across both directions. Scapular values are expressed in millimeters of translation along the thoracic plane. Data for the human shoulder are based on normative kinematic references, whereas AssistOn-Arm values derive from CAD-based kinematic simulation and experimental verification.

**Table 3:** Standardized Movement Tasks Across Anatomical Planes for Device Validation.

Movement Number	Primary Plane/Function	Task Description	Clinical Relevance *	Tested Population
M1	Sagittal plane	Flexion	Fundamental daily reaching	H, P
M2	Sagittal/multi-planar	Elevation—Full upward arm elevation	Overhead activities	H, P
M3	Sagittal plane	Hyperextension	Behind-body reach	H, P
M4	Coronal plane	Abduction–Adduction	Dressing, lateral reach	H, P
M5	Transverse (shoulder 90°)	External Rotation—At 90° abduction, elbow at 90° flexion	Functional stability	H, P
M6	Transverse (neutral arm)	Internal/External Rotation—Arm beside trunk, elbow flexed/extended	Grooming, tool use	H, P
M7	Functional behind-trunk	External Rotation—Hand behind trunk, shoulder at 180° flexion, elbow flexed	Cervical-level reach (C1–C7)	H
M8	Functional behind-trunk	Internal Rotation—Hand behind trunk, shoulder extended, elbow flexed	Lumbar/pelvic reach (L1–sacrum)	H
M9	Horizontal plane	Horizontal Abduction–Adduction	Eating, self-care	H, P

* Clinical relevance column links each task to ADLs. The selected movements reflect key functional tasks of daily living—such as reaching, dressing, grooming, and self-care—providing clinically relevant measures of shoulder mobility and rehabilitation performance. H: Healthy; P: Patient.

**Table 4:** Comparison of Range of Motion (ROM) and Statistical Outcomes Across Movement Tasks in Exoskeleton-Attached and Free Movement Conditions (Patient Active). Bolded values denote the tasks in which kinematic measures differed significantly (*p* < 0.05) between the measurement conditions, reflecting sensitivity of the device to capture task-specific variations.

Movement Number	AssistOn-Arm (°)	Xsens MVN (°)	Free Movement (°)	F (dF)	*p*	Effect Sizes (Cohen’s d [95% CI])
M1	170.7 ± 6.9	160.9 ± 5.7	161.1 ± 5.6	9.01 (86)	**<0.05**	AssistOn-Arm vs. Xsens: 1.11 [0.79–1.63] AssistOn-Arm vs Free: 0.62 [0.31–0.98] Xsens vs. Free: −0.02 [−0.47–0.32]
M2	163.6 ± 5.7	155.5 ± 4.9	164.3 ± 4.9	4.51 (86)	**<0.05**	AssistOn-Arm vs. Xsens: 0.78 [0.51–1.04] AssistOn-Arm vs. Free: 0.30 [0.04–0.83] Xsens vs. Free: −0.48 [−0.81–−0.17]
M3	37.5 ± 1.3	35.6 ± 1.3	40.4 ± 2.4	2.48 (90)	0.09	AssistOn-Arm vs. Xsens: 0.39 [0.03–0.76] AssistOn-Arm vs. Free: −1.06 [−1.59–0.77] Xsens vs. Free: −0.47 [−0.57–0.29]
M4	156.6 ± 1.4	168.1 ± 6.9	169.0 ± 11.5	2.95 (74)	0.06	AssistOn-Arm vs. Xsens: −1.47 [−2.2–1.17] AssistOn-Arm vs. Free: −1.26 [−1.65–−0.38] Xsens vs. Free: −0.45 [−0.38–0.37]
M5	96.2 ± 8.2	94.5 ± 6.5	108.5 ± 10.9	3.84 (89)	**<0.05**	AssistOn-Arm vs. Xsens: −1.08 [−1.6–−0.48] AssistOn-Arm vs. Free: −0.45 [−1.1–−0.04] Xsens vs. Free: 0.35 [0.17–0.99]
M7	178.7 ± 11.0	163.1 ± 8.5	165.2 ± 8.5	14.98 (89)	**<0.05**	AssistOn-Arm vs. Xsens: −2.05 [−3.9–−1.35] AssistOn-Arm vs. Free: −2.52 [−3.48–−2.06] Xsens vs. Free: −1.86 [−2.67–−1.42]
M8	42.3 ± 1.5	40.2 ± 1.5	53.4 ± 7.1	19.59 (77)	**<0.05**	AssistOn-Arm vs. Xsens: −0.08 [−0.47–0.3] AssistOn-Arm vs. Free: −0.55 [−0.85–−0.26] Xsens vs. Free: −0.54 [−0.84–−0.26]
M9	111.6 ± 3.3	107.0 ± 5.2	117.3 ± 5.2	2.59 (74)	0.08	AssistOn-Arm vs. Xsens: 1.09 [0.75–1.6] AssistOn-Arm vs. Free: 0.93 [0.60–1.39] Xsens vs. Free: −0.19 [−0.50–0.18]

Values are presented as mean ± standard deviation (SD). ROM data were obtained under two exoskeleton-attached conditions—Participant-Active (PA: AssistOn-Arm in passive mode, participant moves voluntarily) and Device-Active (DA: AssistOn-Arm in record-and-play mode)—and compared against Free Movement (Xsens MVN only). Columns include descriptive statistics for AssistOn-Arm and Xsens MVN measurements, as well as repeated-measures ANOVA results (Sum of Squares, Mean Square, F, degrees of freedom [df], and significance level [σ]). Significant values (*p* < 0.05) indicate tasks in which kinematics differed significantly between measurement conditions, reflecting sensitivity of the device to capture task-specific variations. Effect sizes are reported as paired Cohen’s d with 95% bootstrap confidence intervals (5000 resamples). Thresholds: small (0.20–0.49), medium (0.50–0.79), large (≥0.80). Note: effect sizes were not separately computed for DA sub-rows.

## Data Availability

The datasets used and analyzed during the current study are available from the corresponding author upon request.

## Supplementary Materials

The following supporting information can be downloaded at: https://www.mdpi.com/article/10.3390/s26010088/s1, Supplementary Material S1. Demonstration and Emergency Procedure Protocol. Supplementary Material S2. AssistOn-Arm Usability and Discomfort Questionnaire.

## Abbreviations

The following abbreviations are used in this manuscript:

ADL	Activities of Daily Living
DoF	Degrees of freedom
ROM	Range of motion
IQR	Interquartile range
