# Supplementary material for: Advancing Precision Rehabilitation Through a Sensor-Based 6-DoF Robotic Exoskeleton: Clinical Validation and Ergonomic Assessment
Source: Sensors (Basel). 2025 Dec 23;26(1):88. doi: 10.3390/s26010088 (PMC12788030; doi:10.3390/s26010088)
Supplement: Supplementary file 1 [file sensors-26-00088-s001.zip › sensors-3942158-supplementary.pdf]

sensors

MDPI

Supplementary Material S1. Demonstration and Emergency Procedure Protocol

Prior to each data collection session, all participants underwent a standardized demonstration and safety briefing lasting approximately 10 minutes. The procedure included the following steps:

1. Introduction and Purpose

• The study objectives and movement tasks were explained verbally.

• Participants were informed that they could stop the session at any time without penalty.

2. Device Demonstration

• Key components of the AssistOn-Arm were introduced, including arm supports, shoulder and elbow modules, and control panel.

• The research member demonstrated each movement mode (Participant-Active and Device-Active) without participant involvement.

3. Safety and Emergency Strategy

• Participants were shown the emergency stop button and instructed on its use.

• The device includes an automatic torque limit that halts motion if resistance exceeds preset safety thresholds.

• During all sessions, researcher(s) remained beside the participant to ensure immediate intervention if discomfort or unexpected motion occurred.

• Testing was discontinued if the participant reported pain, excessive fatigue, or instability.

4. Post-Demonstration Confirmation

• Participants confirmed understanding of the procedures and consented to proceed with testing.

• The entire demonstration was conducted in accordance with the ethical protocol (Acibadem University approval 2018-7/2).

Supplementary Material S2. AssistOn-Arm Usability and Discomfort Questionnaire

The following 13-item questionnaire was used to assess usability, comfort, and perceived safety of the AssistOn-Arm device. Each item was rated on a 5-point Likert scale (1 = strongly disagree, 5 = strongly agree).

No	Questionnaire Item	Strongly Disagree (1)	Disagree (2)	Neutral (3)	Agree (4)	Strongly Agree (5)
Q1	AssistOn-Arm connections were easily installed					
Q2	AssistOn-Arm connections were easily removed					
Q3	AssistOn-Arm connections did not disturb my arm					
Q4	AssistOn-Arm connections did not cause irritation on my skin during use					
Q5	AssistOn-Arm connections were easily adjusted to my arm					
Q6	AssistOn-Arm connections adequately supported my arm					
Q7	The handle of AssistOn-Arm was easy-to-use					
Q8	There was no stress on my movements while I was connected to AssistOn-Arm					
Q9	I was able to move my arm freely to the position I want while moving with AssistOn-Arm					
Q10	I was able to access the emergency stop button of AssistOn-Arm					
Q11	I felt safe and confident during my movements with AssistOn-Arm					
Q12	There was no skin irritation after the tests with AssistOn-Arm					
Q13	I felt no pain in my arm after the tests with AssistOn-Arm					
